# Immune cells mediated the causal relationship between the gut microbiota and lung cancer: a Mendelian randomization study

**DOI:** 10.3389/fmicb.2024.1390722

**Published:** 2024-05-03

**Authors:** Zhiting Chen, Zhe Wang, Hao Ma, Hejing Bao, Ting Jiang, Ting Yang, Shudong Ma

**Affiliations:** ^1^Department of Oncology, Nanfang Hospital, Southern Medical University, Guangzhou, China; ^2^Department of Obstetrics and Gynecology, Shijing People’s Hospital, Guangzhou, China; ^3^Department of Oncology, Panyu Central Hospital, Guangzhou, China

**Keywords:** lung cancer, gut microbiota, immune cell, gut-lung axis, Mendelian randomization

## Abstract

**Introduction:**

The gut microbiota (GM) influences the occurrence and progression of lung cancer (LC), with potential involvement of immune cells (IC). We aimed to investigate the causal impact of GM on LC and identify potential immune cell mediators.

**Methods:**

The utilized data for the Genome-Wide Association Studies (GWAS) were summarized as follows: gut microbiota data from the Dutch Microbiome Project (DMP) (*N* = 7,738), lung cancer data from the Transdisciplinary Research in Cancer of the Lung (TRICL) and International Lung Cancer Consortium (ILCCO) (*N*_*case*_ = 29,266, *N*_*control*_ = 56,450) included four types of cancer: NSCLC, LUAD, LUSC, and SCLC, and immune cell data from European populations (*N* = 3,757). We employed bi-directional two-sample univariable Mendelian randomization (UVMR), multivariable Mendelian randomization (MVMR), and mediation analysis to assess the causal relationship between GM and LC and potential immune cell mediators.

**Results:**

Bi-directional UVMR analysis revealed that 24 gut microbiota species can affect LC, while LC can affect the abundance of 17 gut microbiota species. Mediation analysis demonstrated that six immune cells mediated the causal relationships of seven gut microbiota species on LC: “CCR7 on naive CD8+ T cell” mediated the causal relationship between s_Alistipes_putredinis and LUAD, with a mediation proportion of 9.5% and *P* = 0.018; “IgD− CD27− B cell %lymphocyte” mediated the causal relationships between g_Gordonibacter and s_Gordonibacter_pamelaeae with LUSC, with mediation proportions of 11.8% and 11.9%, respectively and *P* = 0.029; “CD20− CD38− B cell %lymphocyte” mediated the causal relationship between s_Bacteroides_clarus and SCLC, with a mediation proportion of 13.8% and *P* = 0.005; “CD20 on IgD+ CD38− unswitched memory B cell” mediated the causal relationship between s_Streptococcus_thermophilus and SCLC, with a mediation proportion of 14.1% and *P* = 0.023; “HLA DR on CD14− CD16+ monocyte” mediated the causal relationship between s_Bifidobacterium_bifidum and SCLC, with a mediation proportion of 8.7% and *P* = 0.012; “CD45 on Granulocytic Myeloid-Derived Suppressor Cells” mediated the causal relationship between f_Lactobacillaceae and SCLC, with a mediation proportion of 4.0% and *P* = 0.021.

**Conclusion:**

This Mendelian randomization study identified several specific gut microbiotas that exhibit causal relationships with lung cancer and potentially mediate immune cells.

## 1 Introduction

Lung cancer (LC) has long been among the leading causes of cancer incidence and mortality globally, posing a significant threat to human health and safety (Sung et al., 2021). The occurrence and progression of lung cancer are influenced by factors such as environment, genetics, and lifestyle habits. The gut microbiota (GM) plays a crucial role in the progression and outcomes of lung cancer by modulating metabolism, the immune system, and inflammatory factors.

Recent theories suggest that despite the physical segregation and independence of the digestive and respiratory tracts, they exhibit a remarkably high degree of similarity in embryonic origin and structure (Georgiou et al., 2021). The complex bidirectional lymphatic and blood communication between the gut and lung forms the gut-lung axis, sharing components of the mucosal immune system (Budden et al., 2017; Enaud et al., 2020; Zhang D. et al., 2020). Microbial communities in the gut metabolize carbohydrates and enhance levels of short-chain fatty acids (SCFA) (Arrieta et al., 2015), thus shaping the immune environment in the lungs. SCFA activate IL-22, regulatory T cells, and Th2 cells in the lungs via T cell receptor signaling, reducing inflammation and lowering the incidence of lung cancer (Correa et al., 2022). The gut microbiota can activate B cells, T cells, and other immune cells, infiltrating the lungs via the bloodstream or lymphatic pathways. This activation triggers pulmonary immune responses, leading to the onset of pulmonary diseases such as COPD and asthma (Budden et al., 2017). Moreover, the gut microbiota may exert a significant impact on the response to immune checkpoint therapy and chemotherapy in lung cancer (Hakozaki et al., 2020). These studies collectively suggest that bidirectional regulation of the gut-lung axis mediated by the host immune system involves the crucial role of immune cells (ICs). Hence, we hypothesize the existence of causal relationships between GM, IC, and LC. We aim to clarify these associations and identify potential GM and immune targets for early diagnosis and clinical treatment.

Mendelian randomization (MR), using genetic variants as instrumental variables (IVs) to infer causal relationships between exposure and outcome, is a method that controls for potential confounding factors and avoids reverse causation bias (Lawlor et al., 2008). Moreover, an increasing number of Genome-Wide Association Studies (GWAS) have identified human genetic information related to the gut microbiota. Therefore, we employ MR to infer causal relationships between GM and LC and further dissect the associations between GM, IC, and LC.

## 2 Materials and methods

### 2.1 Study design

**FIGURE 1:**
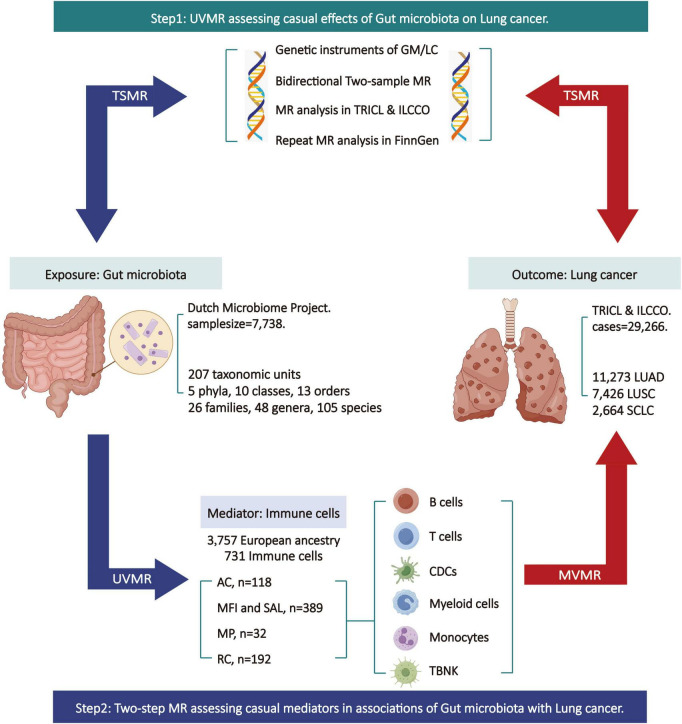
Overview of the Mendelian randomization (MR) study design. AC, absolute cell counts; GM, gut microbiota; LC, lung cancer; LUAD, lung adenocarcinoma; LUSC, squamous cell lung carcinoma; ILCCO, International Lung Cancer Consortium; MFI, median fluorescence intensity; MP, morphological parameters; MR, Mendelian randomization; MVMR, multivariable Mendelian randomization; RC, relative cell counts; SAL, surface antigen levels; SCLC, small cell lung cancer; TBNK, T cells B cells and natural killer cells; TSMR, two sample Mendelian randomization; TRICL, Transdisciplinary Research in Cancer of the Lung; UVMR, univariable Mendelian randomization. This figure was created by Figdraw (www.figdraw.com).

The specific design and workflow of this MR study are divided into two steps (Figure 1). In the first step, bi-directional two-sample univariable Mendelian randomization (UVMR) was used to assess the causal impact of GM on LC, following the three fundamental assumptions of MR analysis (Hingorani and Humphries, 2005): selected SNPs (1) should be closely associated with the exposure, (2) should affect the outcome only through the exposure, and (3) should not be associated with potential confounding factors. Reverse analyses were conducted to eliminate exposures with reverse causal relationships with outcomes. In the second step, both UVMR and multivariable Mendelian randomization (MVMR) analyses were employed to evaluate the mediating role of IC in the pathway between GM and LC, calculating the effect size and proportion for each eligible mediator. This research adheres to the guidelines of Strengthening the Reporting of Observational Studies in Epidemiology using Mendelian Randomization (STRBOE-MR) (Skrivankova et al., 2021; Supplementary Table 1).

### 2.2 Data sources

The gut microbiota data is derived from the Dutch Microbiome Project (DMP), a GWAS conducted on 7,738 individuals of European descent (Lopera-Maya et al., 2022). The data was determined through shotgun metagenomic sequencing of fecal samples, encompassing a total of 207 taxonomic units (5 phyla, 10 classes, 13 orders, 26 families, 48 genera, and 105 species). The immune cell data comes from GWAS summary statistical data containing 731 immune-related whole-genome features (Orru et al., 2020). This data comprised 3,757 individuals of European ancestry without any overlapping cohorts. The immune features encompass absolute cell counts (AC, *n* = 118), median fluorescence intensity reflecting surface antigen levels (MFI and SAL, *n* = 389), morphological parameters (MP, *n* = 32), and relative cell counts (RC, *n* = 192). The MFI, AC, and RC features encompass mature stages of B cells, CDCs, T cells, monocytes, myeloid cells, TBNK (T cells, B cells, and natural killer cells), and Treg panels. The MP features encompass CDC and TBNK panels. Summary of statistical data for two-sample MR analysis and mediation MR analysis was from Transdisciplinary Research in Cancer of the Lung (TRICL) and International Lung Cancer Consortium (ILCCO) (McKay et al., 2017). The data involves aggregated GWAS analyses of lung cancer, comprising 29,266 cases and 56,450 controls. It includes data on three types of lung cancer: lung adenocarcinoma (LUAD) (cases = 11,273, controls = 55,483), squamous cell lung carcinoma (LUSC) (cases = 7,426, controls = 55,627), and small cell lung cancer (SCLC) (cases = 2,664, controls = 21,444). Additionally, validation of the results for the two-sample MR analysis is conducted using lung cancer data (including NSCLC and three pathological subtypes) from FinnGen database. Refer to Table 1 for the specific details of all the data.

**TABLE 1:** Data sources.

Phenotypes	Cases/controls or sample sizes	Data source	Phenotypic code	Ancestry
**Exposure**				
Gut microbiota	7,738	DMP	GCST90027446 to GCST90027857	European
**Mediator**				
Immune cells	3,757	Orru et al., 2020	GCST0001391 to GCST0002121	European
**Outcome**				
NSCLC	29,266/56,450	TRICL and ILCCO	GCST004748	European
LUAD	11,273/55,483	TRICL and ILCCO	GCST004744	European
LUSC	7,426/55,627	TRICL and ILCCO	GCST004750	European
SCLC	2,664/21,444	TRICL and ILCCO	GCST004746	European
**Validation**				
NSCLC	5,315/308,878	FinnGen	C3_LUNG_NONSMALL_EXALLC	European
LUAD	1,590/312,603	FinnGen	C3_NSCLC_ADENO_EXALLC	European
LUSC	1,510/312,683	FinnGen	C3_NSCLC_SQUAM_EXALLC	European
SCLC	717/313,476	FinnGen	C3_SCLC_EXALLC	European

DMP, Dutch Microbiome Project; ILCCO, International Lung Cancer Consortium; TRICL, Transdisciplinary Research in Cancer of the Lung.

### 2.3 Genetic instrumental variable selection

We followed these steps to screen IVs for analysis. Firstly, we set the significance threshold for IVs of GM and IC at 1e−05 and for LC at 5e−08. Secondly, PLINK software (version v1.90) was used to eliminate SNPs with linkage disequilibrium (LD) (*r*^2^ < 0.001 within a 10,000 kb distance) (Auton et al., 2015). Thirdly, SNPs significantly associated with the outcomes (*P*-value < 5e−05) were excluded. Fourthly, we eliminated palindromic SNPs to ensure that the SNP’s effects on exposure and outcome corresponded to the same allele. Finally, we calculated the *F*-statistic values to measure IVs’ strength (Burgess et al., 2019), retaining SNPs with *F*-values values greater than 10 and removing those with a minor allele frequency (MAF) less than 0.01. Detailed information on these IVs is available in Supplementary Table 2.

### 2.4 UVMR and MVMR analysis

In evaluating the causal relationship between the gut microbiota and lung cancer, Inverse Variance Weighted (IVW) is the main method (Lawlor et al., 2008), and the other four methods are used as supplementary analyses (Weighted Median, MR-Egger regression, Weighted Mode, and Sample mode). For binary outcome results, odds ratios (OR) are reported with corresponding 95% confidence intervals, while for continuous outcomes, β-values are reported. In MVMR analysis, the Multivariable Inverse Variance Weighted method (MV-IVW) is employed as the primary analysis. Results with a *P*-value < 0.05 were considered to have a causal relationship. Correction for multiple testing of all IVW results was performed using the false discovery rate (FDR) method, and FDR *q*-values were provided. The results with FDR *q*-value < 0.05 were considered significant.

### 2.5 Mediation MR analysis

**FIGURE 2:**
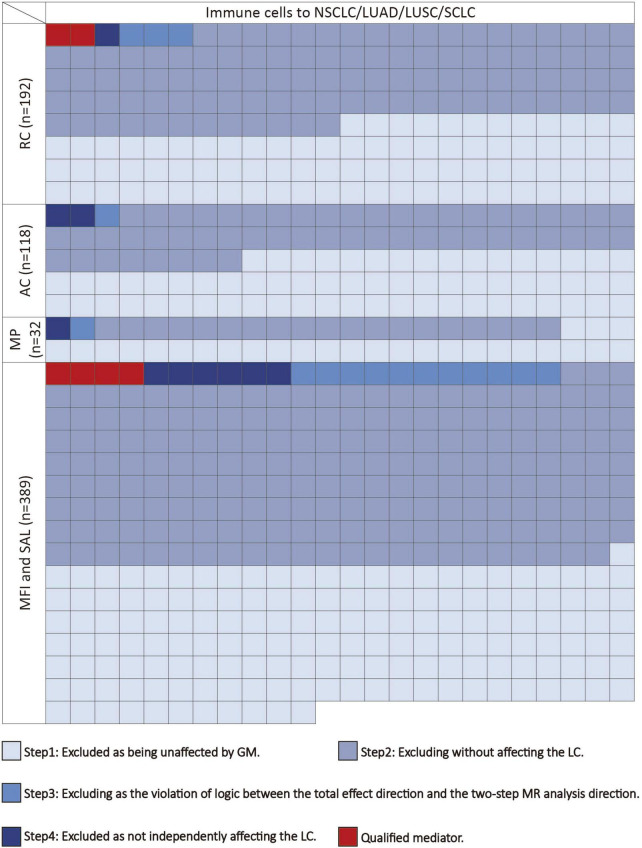
The mediating selection process in the causal relationship between the gut microbiota and lung cancer. AC, absolute cell counts; LC, lung cancer; LUAD, lung adenocarcinoma; LUSC, squamous cell lung carcinoma; MFI, median fluorescence intensity; MP, morphological parameters; RC, relative cell counts; SAL, surface antigen levels; SCLC, small cell lung cancer.

We conducted the following steps to identify potential immune cells mediating the gut microbiota-lung cancer pathway (Figure 2). In the first step, using UVMR, we screened immune cells causally influenced by the gut microbiota and computed their effect values (β1). In the second step, we used UVMR to screen for mediators among the immune cells identified in the first step that causally affect lung cancer, also computing their effect values (α). In the third step, based on the directions of the effect values obtained for the gut microbiota-lung cancer (β), gut microbiota-immune cells (β1), and immune cells-lung cancer (α) pathways, we retained logically consistent mediators (if the effect size β for the gut microbiota’s total effect on lung cancer is positive, then β1 and α should be both positive or negative; conversely, if the effect size β for the gut microbiota’s total effect on lung cancer is negative, then β1 and α should have one positive and one negative). In the fourth step, for the immune cells obtained from the first three steps, we used MVMR to evaluate the causal impact of these immune cells on lung cancer after adjusting for gut microbiota (β2), thereby selecting immune cells independently acting on the outcome. Subsequently, combining the gut microbiota’s causal effect on lung cancer obtained from UVMR (β), we used the “product of coefficients” method to calculate the mediated effect of immune cells in the gut microbiota-lung cancer pathway (β1 × β2) and the proportion of the mediated effect ([β1 × β2] / β) (Burgess et al., 2019).

### 2.6 MR sensitivity analysis

We conducted sensitivity analyses using MR Egger regression, leave-one-out method, and MR-PRESSO method. Cochran’s *Q* statistic was calculated for each SNP to assess heterogeneity, and the *P*-value from the intercept test of MR Egger regression was used to evaluate horizontal pleiotropy (Bowden et al., 2015). The MR-PRESSO method was employed to correct for potential horizontal pleiotropy of the selected IVs (Verbanck et al., 2018). P_heterogeneity < 0.05 was considered indicative of heterogeneity, while P_Global.test and P_pleiotropy < 0.05 were considered indicative of pleiotropy. Evidence of pleiotropy would invalidate causality.

All analyses were conducted using the TwoSampleMR (Hemani et al., 2018), MR-PRESSO (Verbanck et al., 2018), and MendelianRandomization (Yavorska and Burgess, 2017) packages in R software (version 4.3.0).

## 3 Results

### 3.1 Genetic instruments for exposures

Following the above steps, we selected IVs for GM, IC, and LC. The number of SNPs for GM ranged from 4 to 59 (median = 28), for IC ranged from 10 to 217 (median = 23), and for LC ranged from 25 to 58 (median = 41). Additionally, all SNPs had an *F*-statistic greater than 15, indicating the use of strong instrumental variables (Supplementary Table 2).

### 3.2 Causal associations of gut microbiota with lung cancer

**FIGURE 3:**
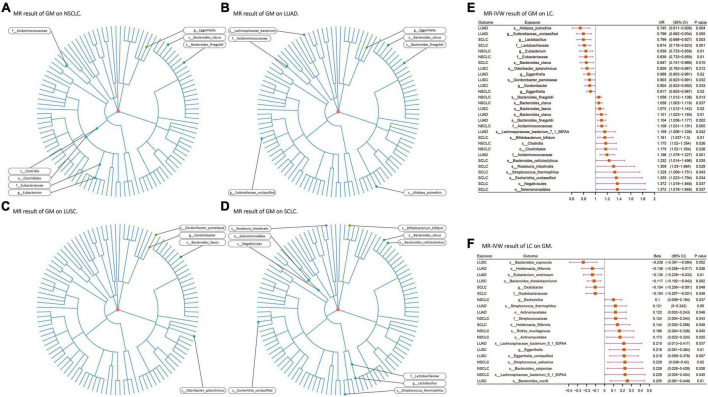
**(A–D)** Various microorganisms within the gut microbiota exhibiting a causal relationship with four distinct types of lung cancer. **(E,F)** Results of bi-directional UVMR on the interplay between the gut microbiota and lung cancer. GM, gut microbiota; LC, lung cancer; LUAD, lung adenocarcinoma; LUSC, squamous cell lung carcinoma; SCLC, small cell lung cancer. The prefix “c_/o_/f_/g_/s_” represents class/order/family/genus/species, respectively.

Among the 207 GM species included in the analysis, ultimately, 24 gut microbiota species (2 orders, 2 families, 3 genera, 5 species from p_Actinobacteria, p_Bacteroidetes, p_Firmicutes, and p_Proteobacteria) were found to have causal relationships with 4 types of lung cancer (Figures 3A–D). When assessing the causal effects of GM on NSCLC using IVW as the primary method, it was found that f_Eubacteriaceae (OR = 0.838; *P* = 0.01), g_Eubacterium (OR = 0.838; *P* = 0.01), and g_Eggerthella (OR = 0.917; *P* = 0.02) were negatively associated with the risk of NSCLC, while c_Clostridia (OR = 1.175; *P* = 0.02), o_Clostridiales (OR = 1.175; *P* = 0.02), f_Acidaminococcaceae (OR = 1.108; *P* = 0.005), s_Bacteroides_clarus (OR = 1.058; *P* = 0.036), and s_Bacteroides_finegoldii (OR = 1.057; *P* = 0.01) were positively associated with the risk of NSCLC. Subsequently, the causal effects of GM on the other three pathological subtypes of lung cancer were evaluated. In LUAD, 7 gut microbiota species (including 1 phylum, 2 families, and 4 species from p_Actinobacteria, p_Bacteroidetes, p_Firmicutes, and p_Proteobacteria) were found to be causally related to LUAD, with f_Acidaminococcaceae (OR = 1.197; *P* = 0.0007) having the strongest impact on LUAD. In LUSC, 4 gut microbiota species (including 1 genus and 3 species from p_Actinobacteria and p_Bacteroidetes) were found to be causally related to LUSC, with s_Odoribacter_splanchnicus (OR = 0.859; *P* = 0.011) having the strongest impact on LUSC. In SCLC, 10 gut microbiota species (including 1 class, 1 order, 1 family, 1 genus, and 6 species from p_Actinobacteria, p_Bacteroidetes, p_Firmicutes, and p_Proteobacteria) were found to be causally related to SCLC, with f_Lactobacillaceae (OR = 0.814; *P* = 0.001) having the strongest impact on SCLC (Figure 3E). In the analysis with FinnGen data as the outcome and IVW as the primary analysis method to verify that f_Acidaminococcaceae (OR = 1.185; *P* = 0.005) was positively correlated with NSCLC risk, f_Lactobacillaceae (OR = 0.806; *P* = 0.029) was negatively correlated with SCLC risk. Detailed results of all UVMR analysis are presented in Supplementary Table 3.

When assessing the causal effects of LC on the GM using IVW as the primary method, it was found that four types of lung cancer could influence the abundance of 17 gut microbiota species (including 1 order, 2 families, 3 genera, and 11 species from p_Actinobacteria, p_Bacteroidetes, p_Firmicutes, and p_Proteobacteria). In NSCLC, the abundance of s_Streptococcus_salivarius (β = 0.227; *P* = 0.02) was most strongly affected. In LUAD, the abundance of s_Eubacterium_ventriosum (β = −0.134; *P* = 0.01) was most strongly affected, and the results passed multiple testing correction (FDR-q: 0.04). In LUSC, the abundance of s_Bacteroides_thetaiotaomicron (β = −0.116; *P* = 0.002) was most strongly affected, and the results for the five gut microbiota species affected by LUSC all passed multiple testing correction. In SCLC, the abundance of s_Holdemania_filiformis (β = 0.143; *P* = 0.047) was most strongly affected (Figure 3F). No exposure and outcome with directional causal relationships were found in the reverse analysis. Detailed results of all UVMR analysis are presented in Supplementary Table 3.

### 3.3 Mediation analyses of potential immune cells

**FIGURE 4:**
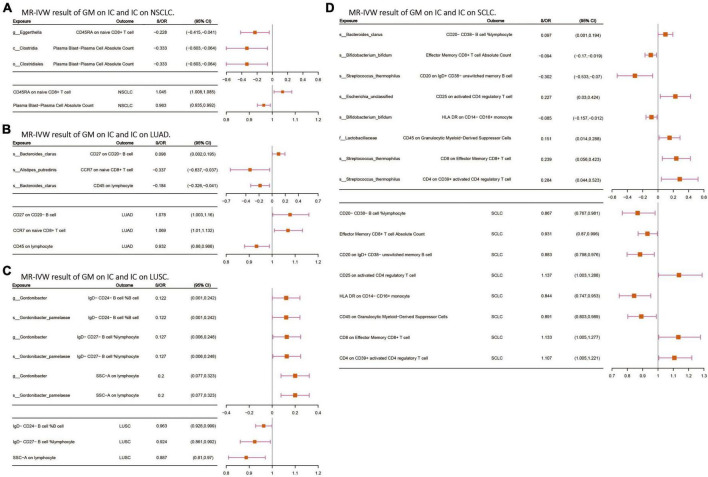
**(A–D)** The MR-IVW analysis results of the interplay between the gut microbiota and immune cells, with each independently impacting four distinct types of lung cancer. GM, gut microbiota; LC, lung cancer; LUAD, lung adenocarcinoma; LUSC, squamous cell lung carcinoma; SCLC, small cell lung cancer. The prefix “c_/o_/f_/g_/s_” represents class/order/family/genus/species, respectively.

**FIGURE 5:**
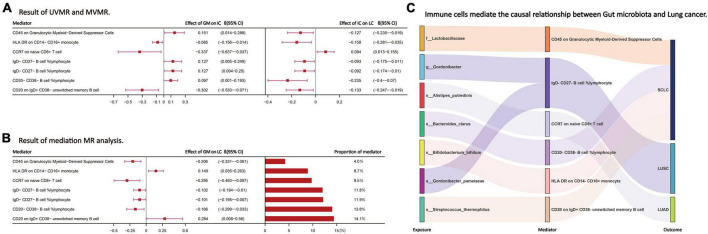
**(A,B)** The effect values and proportions of each intermediate immune cell phenotype in the causal relationship between the gut microbiota and lung cancer. **(C)** Through the above steps, the intermediate immune cell phenotype selected to mediate the causal relationship between the GM and LC. LC, lung cancer; LUAD, lung adenocarcinoma; LUSC, squamous cell lung carcinoma; SCLC, small cell lung cancer. The prefix “f_/g_/s_” represents family/genus/species, respectively.

Following the screening of potential immune cell phenotypes, a total of six immune cell phenotypes met our selection criteria. Initially, through two-step UVMR and directional screening of β, β1, and α, we preliminarily identified 20 gut microbiota-immune cell-lung cancer pathways, encompassing 16 immune cell phenotypes (Figures 4A–D and Supplementary Tables 4, 5). Subsequently, in the fourth step, we used MVMR to select immune cells that could independently affect on lung cancer and computed the values of mediated effects and proportions of immune cell mediation. After adjusting for the influence of gut microbiota and calculating the mediating effect values and proportions, we found that “CCR7 on naive CD8+ T cell” exhibited mediation in the causal association between s_Alistipes_putredinis and LUAD, with a mediation proportion of 9.5% (*P* = 0.018); “IgD− CD27− B cell %lymphocyte” showed mediation in the causal associations between g_Gordonibacter and s_Gordonibacter_pamelaeae with LUSC, with mediation proportion s of 11.8% and 11.9%, respectively (*P* = 0.029); “CD20− CD38− B cell %lymphocyte” demonstrated mediation in the causal association between s_Bacteroides_clarus and SCLC, with a mediation proportion of 13.8% (*P* = 0.005); “CD20 on IgD+ CD38− unswitched memory B cell” displayed mediation in the causal association between s_Streptococcus_thermophilus and SCLC, with a mediation proportion of 14.1% (*P* = 0.023); “HLA DR on CD14− CD16+ monocyte” exhibited mediation in the causal association between s_Bifidobacterium_bifidum and SCLC, with a mediation proportion of 8.7% (*P* = 0.012); and “CD45 on Granulocytic Myeloid-Derived Suppressor Cells” showed mediation in the causal association between f_Lactobacillaceae and SCLC, with a mediation proportion of 4.0% (*P* = 0.021) (Table 2, Supplementary Table 7, and Figures 5A–C).

**TABLE 2:** Multivariable Mendelian randomization estimates for the causal associations of IC with LC with adjustment for GM.

Outcome	Mediator	OR (95% CI)	IVW *P*-value	Adjust for	β (95% CI)	MV-IVW *P*-value
**UVMR analysis**				**MVMR analysis**		
NSCLC	D45RA on naive CD8+ T cell	1.045 (1.008, 1.085)	0.020	g_Eggerthella	0.048 (−0.007, 0.102)	0.086
NSCLC	Plasma blast-plasma cell absolute count	0.963 (0.935, 0.992)	0.012	c_Clostridia	−0.054 (−0.128, 0.019)	0.148
NSCLC	Plasma blast-plasma cell absolute count	0.963 (0.935, 0.992)	0.012	o_Clostridiales	−0.054 (−0.128, 0.019)	0.148
LUAD	CD27 on CD20− B cell	1.078 (1.003, 1.160)	0.042	s_Bacteroides_clarus	0.074 (−0.035, 0.183)	0.182
LUAD	CCR7 on naive CD8+ T cell	1.069 (1.010, 1.132)	0.022	s_Alistipes_putredinis	0.084 (0.015, 0.154)	0.018
LUAD	CD45 on lymphocyte	0.932 (0.880, 0.988)	0.017	s_Bacteroides_clarus	−0.022 (−0.084, 0.041)	0.499
LUSC	IgD− CD24− B cell %B cell	0.963 (0.928, 0.999)	0.046	g_Gordonibacter	−0.099 (−0.204, 0.005)	0.062
LUSC	IgD− CD24− B cell %B cell	0.963 (0.928, 0.999)	0.046	s_Gordonibacter_pamelaeae	−0.099 (−0.203, 0.005)	0.063
LUSC	IgD− CD27− B cell %lymphocyte	0.924 (0.861, 0.992)	0.028	g_Gordonibacter	−0.092(−0.176, −0.009)	0.029
LUSC	IgD− CD27− B cell %lymphocyte	0.924 (0.861, 0.992)	0.028	s_Gordonibacter_pamelaeae	−0.093 (−0.175, −0.010)	0.029
LUSC	SSC-A on lymphocyte	0.887 (0.810, 0.970)	0.009	g_Gordonibacter	−0.035 (−0.146, 0.076)	0.532
LUSC	SSC-A on lymphocyte	0.887 (0.810, 0.970)	0.009	s_Gordonibacter_pamelaeae	−0.035 (−0.146, 0.076)	0.531
SCLC	CD20− CD38− B cell %lymphocyte	0.867 (0.767, 0.981)	0.023	s_Bacteroides_clarus	−0.235 (−0.400, −0.071)	0.005
SCLC	Effector memory CD8+ T cell absolute count	0.931 (0.870, 0.996)	0.039	s_Bifidobacterium_bifidum	0.032 (−0.069, 0.134)	0.533
SCLC	CD20 on IgD+ CD38− unswitched memory B cell	0.883 (0.798, 0.976)	0.015	s_Streptococcus_thermophilus	−0.133 (−0.247, −0.019)	0.023
SCLC	CD25 on activated CD4 regulatory T cell	1.137 (1.003, 1.288)	0.044	s_Escherichia_unclassified	0.083 (−0.078, 0.244)	0.311
SCLC	HLA DR on CD14− CD16+ monocyte	0.844 (0.747, 0.953)	0.006	s_Bifidobacterium_bifidum	−0.158 (−0.282, −0.035)	0.012
SCLC	CD45 on granulocytic myeloid-derived suppressor cells	0.891 (0.803, 0.989)	0.030	f_Lactobacillaceae	−0.127 (−0.235, −0.019)	0.021
SCLC	CD8 on effector memory CD8+ T cell	1.133 (1.005, 1.277)	0.041	s_Streptococcus_thermophilus	0.092 (−0.077, 0.260)	0.286
SCLC	CD4 on CD39+ activated CD4 regulatory T cell	1.107 (1.005, 1.221)	0.040	s_Streptococcus_thermophilus	0.067 (−0.102, 0.236)	0.437

GM, gut microbiota; LC, lung cancer; MVMR, multivariable Mendelian randomization.

### 3.4 MR sensitivity analysis

In the analyses of GM-LC, LC-GM, and IC-LC, both the MR-Egger intercept and MR-PRESSO *P*-values were greater than 0.05, indicating the absence of minimal horizontal pleiotropy (Supplementary Table 6). However, in the GM-IC analysis, four causal relationships with horizontal pleiotropy were detected (Supplementary Table 6) and subsequently excluded from our final results. In LC-GM, IC-LC, and GM-IC analyses, some IVs showed potential heterogeneity, but instrumental variable strength testing indicated the adequacy of these IVs (all *F*-statistics ≥ 15; Supplementary Tables 2, 6), and no horizontal pleiotropy was detected (all P_Global.test and P_pleiotropy > 0.05; Supplementary Table 6). Leave-one-out analysis demonstrated that in GM-LC and LC-GM results, no individual SNP significantly altered the causal effects (Supplementary Figures 1, 2). Overall, sensitivity analysis confirmed the reliability of the MR results.

## 4 Discussion

In this large-scale MR study, we identified a total of 29 causative relationships with a genetic predisposition between 24 gut microbiota species (4 from p_Actinobacteria, 6 from p_Bacteroidetes, 12 from p_Firmicutes, and 2 from p_Proteobacteria) and lung cancer and its subtypes (8 with NSCLC, 7 with LUAD, 4 with LUSC, and 10 with SCLC). Similarly, lung cancer and its subtypes exhibited genetic predisposition effects on 20 causative relationships with 17 gut microbiota species (4 from p_Actinobacteria, 4 from p_Bacteroidetes, 6 from p_Firmicutes, and 3 from p_Proteobacteria). Moreover, utilizing UVMR and MVMR as mediation analysis methods, we discovered that seven microbial taxa exerted their effects on lung cancer through mediation by six immune cell phenotypes (from B cell panel, Maturation stages of T cell panel, TBNK panel, and Monocyte panel).

The gut microbiota, often referred to as the “second endocrine organ,” can influence the host’s metabolism, inflammation, and other physiological and pathological processes through the production of metabolites and specific small molecules (Miyauchi et al., 2020; Erny et al., 2021).

The final findings of this study primarily focus on the relationship between the gut microbiota, immune cells, and lung cancer, particularly in SCLC. The research indicates that there are significant differences in the immune microenvironment between SCLC and NSCLC. Compared to LUAD, SCLC exhibits greater immune exclusion and less immune infiltration (Chan et al., 2021). Therefore, exploring the mediators through which the gut microbiota influences the tumor microenvironment in SCLC is crucial for identifying potential therapeutic targets. Our research revealed that the immune cell phenotype “CD45 on Granulocytic Myeloid-Derived Suppressor Cells” mediates the effect of f_Lactobacillaceae on SCLC. Lactobacillus is recognized as an immunomodulatory probiotic (Mu et al., 2018), demonstrated in both animal studies and clinical trials to alleviate respiratory symptoms, including inhibiting tumor growth and metastasis to the lungs (Zhang M. et al., 2020), while also reducing chemotherapy side effects (Mendez et al., 2021). “CD45 on Granulocytic Myeloid-Derived Suppressor Cells” represents the expression of myeloid-derived suppressor cells (MDSCs) in peripheral blood, which are a heterogeneous population of immune-suppressive cells derived from the myeloid lineage. Accumulation of granulocytic myeloid-derived suppressor cells from the myeloid lineage weakens anti-tumor T cell responses and diminishes responsiveness of mouse tumors to PD-1 blockade (Li et al., 2021). These findings suggest that f_Lactobacillaceae may influence the expression of myeloid-derived suppressor cells in the body via immune responses, thereby exerting its effect on SCLC. We also found that the immune cell phenotype “HLA DR on CD14− CD16+ monocytes” mediates the effect of s_Bifidobacterium_bifidum on SCLC. Bifidobacterium, as a probiotic, has been reported as a potential biomarker for cancer (Zhuang et al., 2019) and has shown protective effects against oxidative stress-induced DNA damage *in vitro*, thereby preventing cancer (Bhatt et al., 2017). In a mouse study, Bifidobacterium enhanced the production of IFN-γ by enhancing the synthesis of immune-stimulating molecules and metabolic products, triggering an anti-tumor host immune response (Lee et al., 2021). “HLA DR on CD14− CD16+ monocytes” represents the expression of monocytes in peripheral blood, where CD14+ CD16− HLA DR monocytes have been reported as predictive factors for the response to anti-PD-1 immunotherapy in metastatic melanoma (Krieg et al., 2018). Considering IFN-γ as a positive stimulatory factor in immune responses, along with our analysis results, s_Bifidobacterium_bifidum may play a role in stimulating IFN-γ production, thereby enhancing the host’s anti-tumor immune response. Furthermore, the immune cell phenotype “CD20 on IgD+ CD38− unswitched memory B cells” mediates the effect of s_Streptococcus_thermophilus on SCLC. Memory B cells, a subtype of B cells, enhance antibody-mediated immune responses upon secondary infection. Memory B cells are highly enriched in lung cancer tissues and increase somatic mutation frequency (Hao et al., 2022). Streptococcus, as an oral resident bacterium, increases in abundance in the lower respiratory tract of lung cancer patients, associated with upregulation of extracellular signal-regulated kinase (ERK) and phosphoinositide 3-kinase (PI3K) signaling pathways (Tsay et al., 2018). The PI3K signaling pathway is a critical pathway in the cancer development process. Therefore, we can predict that s_Streptococcus_thermophilus alters the expression of memory B cells through the PI3K signaling pathway, thereby influencing the immune microenvironment of SCLC.

In NSCLC and its subtypes LUAD and LUSC with relatively “mild” immune microenvironments, we also identified the “gut microbiota-immune cells-lung cancer” relationship. Firstly, in LUAD, the immune cell phenotype “CCR7 on naive CD8+ T cells” mediates the effect of s_Alistipes_putredinis on LUAD. CCR7 belongs to the anti-tumor chemokine family, guiding dendritic cells (DCs) to migrate to lymphoid organs to initiate immune responses, activate the effector function of T cells, and exhibit anti-tumor activity (Villablanca et al., 2010). In a study investigating the relationship between gut microbiota and PD1 immunotherapy in Chinese NSCLC patients, it was found that patients who responded to the therapy had higher abundance of s_Alistipes_putredinis in their gut. Flow cytometry also showed a higher frequency of unique memory CD8+ T cells and natural killer cell subsets in the peripheral blood of responsive patients (Jin et al., 2019). We can thus predict the possible relationship between s_Alistipes_putredinis, CD8+ T cells, and LUAD. Additionally, both g_Gordonibacter and s_Gordonibacter_pamelaeae exert their effects on LUSC through the immune cell phenotype “IgD− CD27− B cell %lymphocyte,” and IgD− CD27− B cells have been shown to be abundant in lung tumors (Belderbos et al., 2024). Although the intermediate substances obtained in this study have undergone rigorous steps and screening through methods like MR, which effectively avoid bias and pleiotropy, the final effect size accounts for approximately 5%–15% of the total effect size. Considering that biological processes are complex, extensive research is still needed to prove the specific mechanisms discussed above. The intermediate substances obtained in this study may provide some reference value for targeted therapy of gut microbiota and immune substances in future lung cancer treatment.

Strengths of this study: Firstly, a comprehensive GWAS dataset with a large sample size, including summarized data from GM, IC, and LC analyses, has been employed. This approach not only yields a wealth of results but also ensures the robust statistical power of the findings. Secondly, a meticulously designed analytical framework has been implemented to explore the causal relationship between GM and LC. Utilizing methods such as UVMR and MVMR, this investigation has identified six immune cell phenotypes as mediators in the causal pathway from GM to LC. Finally, a variety of MR analysis methods have been employed for causal inference, and sensitivity tests have been conducted to confirm the robustness of the results. This ensures that the findings are resilient, unaffected by horizontal pleiotropy, and not influenced by other factors.

Limitations of this study: firstly, the causal relationship between GM and LC identified through UVMR and MVMR analyses assumes linearity, whereas in reality, this relationship may be more complex, involving environmental factors and other genetic elements. Additionally, the study population primarily consists of individuals of European descent, limiting the generalizability of the results.

## 5 Conclusion

In conclusion, this study comprehensively assessed the causal relationship between gut microbiota, immune cells, and lung cancer. These findings underscore and elucidate potential mechanisms between gut microbiota and lung cancer, offering new insights for immunotherapeutic interventions based on the gut microbiome for lung cancer and immune cell-targeted interventions.

## Data availability statement

All the analytical data in this study are included in the tables and Supplementary material of the article. The raw data for LC, GM, and IC can all be obtained from the GWAS Catalog (https://www.ebi.ac.uk/gwas/).

## Ethics statement

The data used in our MR analysis were obtained entirely from previously reported summary data. Therefore, neither patient consent nor ethical approval were necessary for this study.

## Author contributions

ZC: Conceptualization, Formal analysis, Resources, Software, Visualization, Writing – original draft, Writing – review & editing. ZW: Conceptualization, Formal analysis, Resources, Software, Visualization, Writing – original draft, Writing – review & editing. HM: Conceptualization, Formal analysis, Resources, Software, Visualization, Writing – original draft, Writing – review & editing. HB: Data curation, Investigation, Writing – original draft. TJ: Validation, Writing – original draft. TY: Methodology, Writing – original draft. SM: Funding acquisition, Project administration, Supervision, Writing – review & editing.
