# Supplementary figures and images for: Immune cells mediated the causal relationship between the gut microbiota and lung cancer: a Mendelian randomization study
Source: Front Microbiol. 2024 May 3;15:1390722. doi: 10.3389/fmicb.2024.1390722 (PMC11099228; doi:10.3389/fmicb.2024.1390722)

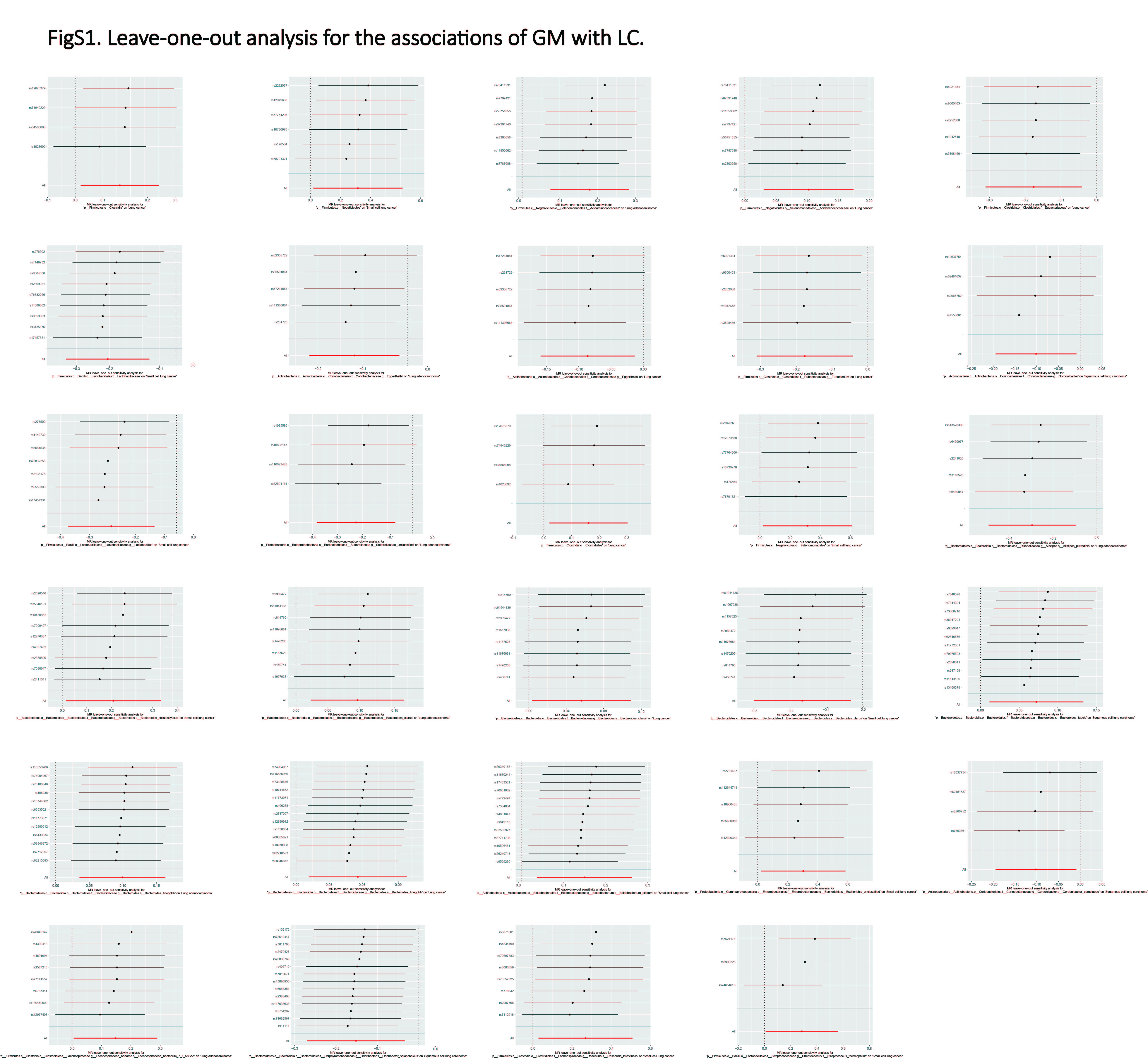

Supplement: Supplementary file 2 [file Image_1.JPEG]

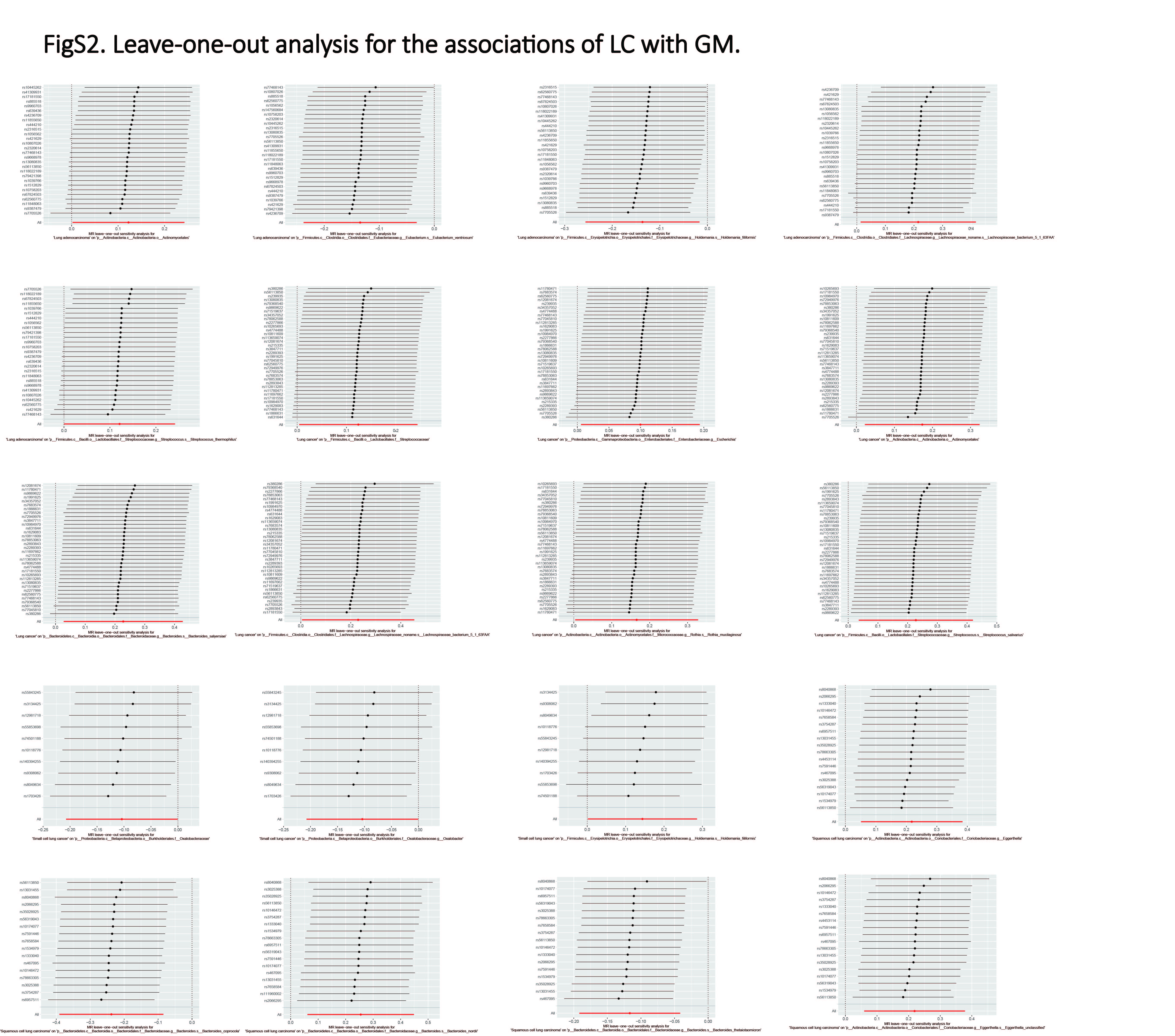

Supplement: Supplementary file 3 [file Image_2.JPEG]
